# Don’t Overweight Weights: Evaluation of Weighting Strategies for Multi-Task Bioactivity Classification Models

**DOI:** 10.3390/molecules26226959

**Published:** 2021-11-18

**Authors:** Lina Humbeck, Tobias Morawietz, Noe Sturm, Adam Zalewski, Simon Harnqvist, Wouter Heyndrickx, Matthew Holmes, Bernd Beck

**Affiliations:** 1 Medicinal Chemistry Department, Boehringer Ingelheim Pharma GmbH & Co. KG, Birkendorfer Str. 65, 88397 Biberach an der Riss, Germany; bernd.beck@boehringer-ingelheim.com; 2 Bayer AG, Pharmaceuticals, R&D, Digital Technologies, Computational Molecular Design, 42096 Wuppertal, Germany; tobias.morawietz@bayer.com; 3 Novartis Institutes for BioMedical Research, CH-4002 Basel, Switzerland; noe.sturm@novartis.com; 4 Amgen Research (Munich) GmbH, Staffelseestraße 2, 81477 Munich, Germany; azalewsk@amgen.com; 5 Computational Sciences, GlaxoSmithKline, Gunnels Wood Road, Stevenage SG1 2NY, UK; seh589@york.ac.uk (S.H.); mwh35@bath.ac.uk (M.H.); 6 Janssen Pharmaceutica N.V., Turnhoutseweg 30, 2340 Beerse, Belgium; wheyndri@its.jnj.com

**Keywords:** machine learning, classification, multi-task learning, federated, weighting, drug design, small molecule drug discovery, MELLODDY

## Abstract

Machine learning models predicting the bioactivity of chemical compounds belong nowadays to the standard tools of cheminformaticians and computational medicinal chemists. Multi-task and federated learning are promising machine learning approaches that allow privacy-preserving usage of large amounts of data from diverse sources, which is crucial for achieving good generalization and high-performance results. Using large, real world data sets from six pharmaceutical companies, here we investigate different strategies for averaging weighted task loss functions to train multi-task bioactivity classification models. The weighting strategies shall be suitable for federated learning and ensure that learning efforts are well distributed even if data are diverse. Comparing several approaches using weights that depend on the number of sub-tasks per assay, task size, and class balance, respectively, we find that a simple sub-task weighting approach leads to robust model performance for all investigated data sets and is especially suited for federated learning.

## 1. Introduction

Drug discovery is a costly and risky (due to high failure rates) endeavor. Time and money spent until market access are continuously increasing for various reasons, including the early-stage effort needed to discover and optimize suitable drug candidates. To overcome this, new avenues are pursued. One particularly promising approach is collaborative efforts between otherwise competing companies, e.g., Martin and Zhu [1], leveraging artificial intelligence (AI) methods [2,3]. Here, we describe a part of the MELLODDY project, a collaborative effort of different pharma companies (referred to as “partner” throughout this article) in the field of multi-task learning [4]. The goal of the project is to train and utilize a federated multi-task feedforward neural network while still preserving the privacy of highly confidential and competitive data. It is an exciting and innovative approach to overcome the stagnation of machine learning model quality and to accelerate drug design that is realized for the first time to a scale this large in drug discovery. Because of the unprecedented scale of the project (increasing from >60 million datapoints in >97,000 tasks in the first year) many fundamental questions and challenges have to be addressed. In general, multi-task models have been shown to be beneficial in drug discovery [5,6,7,8]. Furthermore, increasing the amount of (diverse) high quality data is supposed to increase model performance and applicability domains [1,5,9]. Nevertheless, detailed investigations to leverage the full potential of the new federated multi-task learning approach are needed. Particularly, the diversity, e.g., size and chemical space (applicability domain) of the data sets of the 10 partners within MELLODDY is interesting and challenging. Thus, weighting, i.e., scaling the contribution of each task’s loss to the total loss, the tasks in federated multi-task learning should be considered to mitigate the risk of the learning being dominated by either a small subset of data sets or tasks and to ensure proper distribution of learning efforts. In this work, we focus on discussing and analyzing different weighting strategies for multi-task bioactivity classification models.

A standard approach in multitask learning is to weight all tasks with a constant and equal weight, i.e., no application of a dedicated weighting scheme. However, an equal weight does not necessarily reflect the underlying circumstances, e.g., difficulty of training, relevance of the task, or scale of the task’s loss [10]. Furthermore, the concrete setting in the studied federated machine learning approach (MELLODDY) contains multiple binary classification tasks belonging to one pharmaceutically relevant assay. This means that for each assay the data are binary classified by one to five thresholds (e.g., 1 µM and 10 µM thresholds for an IC50 assay leading to two separate tasks). Within this study, we used two thresholds for each assay: (1) median; and (2) lower quartile if a minimal number (25) of positives and negatives remained. This relationship between tasks wouldn’t be reflected by an equal weight and could lead to a domination of learning by easy tasks, because the machine learning algorithm is rewarded equally for both. In other works, the weights are learned and dynamically adapted during training [11,12,13]. Other approaches use similarities of tasks [14] or a multi-objective optimization view [15] to tune gradients in multi-task learning. In this work, fixed and continuous weighting schemes are analyzed (see Table 1). In the fixed scheme all tasks with more datapoints than a given cutoff are down-weighted. In contrast, the continuous weighting schemes down-weight either dependent on the task size (datapoints), task number (sub-tasks per assay), task classification label balance, or the task fraction of a positive sample (actives, short: fraction actives). Task size dependent weighting was investigated due to the influence of task size on two crucial parameters: difficulty (smaller tasks are assumed to be more difficult) and relevance (larger tasks are not expected to gain much in the federated setting and are hence of less interest [7]). Other works [16] showed superior performance when smaller tasks were upweighted. The two task characteristics, label balance and fraction of actives, are also assumed to be related to task difficulty. Moreover, baseline weighting all tasks equally (all tasks have a weight of 1, “1” in Table 1) is used and a weighting based on task number is applied. A detailed overview of the examined weighting schemes is given in Table 1.

This work is structured in three Results and Discussion sections, which we call phases:

1. Phase I: exploration results from the fixed scheme are discussed.
2. Phase II: results from the continuous scheme and of further fixed scheme experiments are discussed.
3. Phase III: results from weighing experiments based on fraction active or label balance are discussed.

## 2. Results and Discussion

**Figure 1:**
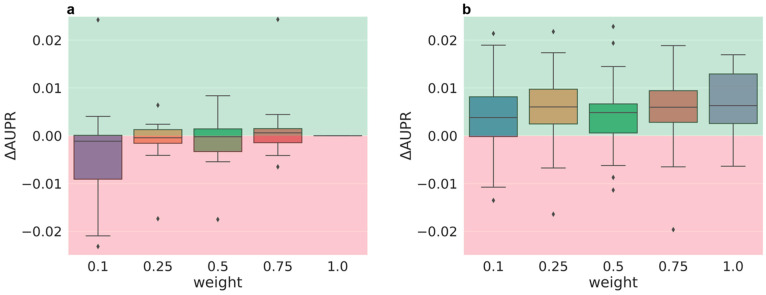
Phase I delta performances (AUPR) of a fixed weighting scheme with threshold 1000 and different weights averaged over 5 partners and 5 folds. (**a**) Synoptic performance (median and lower quartile tasks) and (**b**) sole quartile task performance compared to the synoptic performance of a weight of 1/task_number, where (**a**) both or (**b**) only quartile tasks are considered. Weight of 1.0 in x-axis labels is equal to 1/task_number. Green: better performance than 1/task_number, red: worse performance.

In three phases, several weighting schemes were analyzed (see Table 1). In order to compare their performance, delta performances (measured as AUPR or AUROC) were computed relative to the baseline (equal weight of 1). The thresholding scheme (to divide an assay into classification tasks) applied in all presented weighting studies has two thresholds: the median and the lower quartile (25/75), because this scheme was superior when compared to a thresholding solely based on either median or lower quartile alone (see Figure 1b). The results of detailed analyses on thresholding schemes will be published elsewhere. During the first phase, a fixed weighting scheme was analyzed. The weighting was only dependent on the task size.

### 2.1. Phase I

In phase I, four different weights (0.1, 0.25, 0.5, 0.75) for a fixed weighting scheme where all tasks with more than 1000 datapoints are down-weighted were evaluated based on a subset of all tasks. Overall, the delta AUPR values are small, in the region of −0.02 and 0.01 (see Figure 1a). A slight trend toward higher weights (less down-weighting) performing better (positive delta performances) than lower weights can be observed. Figure 1b shows the benefit of using a quartile and median threshold over only applying a quartile threshold.

### 2.2. Phase II

In phase II the following approaches were investigated: (a) two continuous schemes with steps 500 and 1000 both with a weight of 0.02, fixed weighting schemes with weights 0.6 and 0.9 and (b) cutoff 1000 and (c) cutoff at the 95% quantile of the task size (called cutoff auto) as well as (d) weighting based on the number of tasks and based on the task size. These cutoffs, steps and weights were selected because they performed best in a pretest executed by one partner (weighting schemes analyzed in the pretest are listed in Table S1 in the Supplementary Materials).

#### Quartile Task Performance

**Figure 2:**
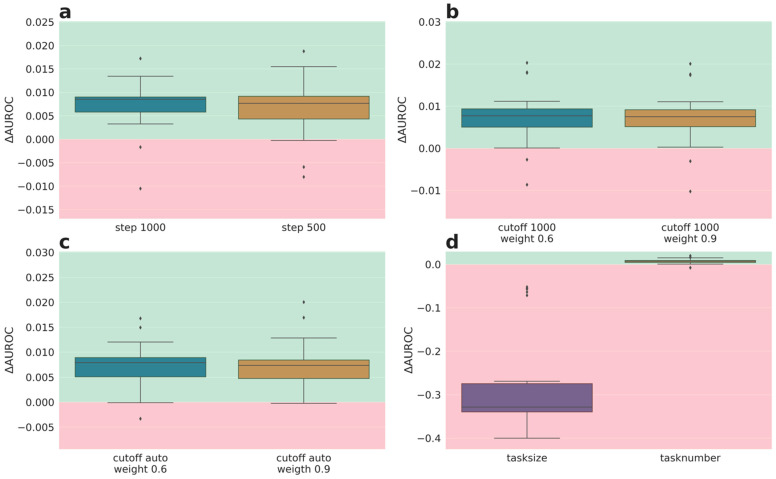
Phase II results of different weighting schemes averaged over 5 partners and 5 folds for lower quartile task (AUROC): (**a**) continuous weighting scheme with weight 0.02 and steps left: 1000 and right: 500, (**b**) fixed weighting scheme with cutoff 1000 and left: weight of 0.6 and right: weight of 0.9, (**c**) fixed weighting scheme with 95% quantile cutoff and left: weight 0.6 and right: weight 0.9, (**d**) left: weighting based on task size, right: weight set to one divided by number of datapoints. Green: better performance than baseline (1), red: worse performance than baseline.

**Figure 3:**
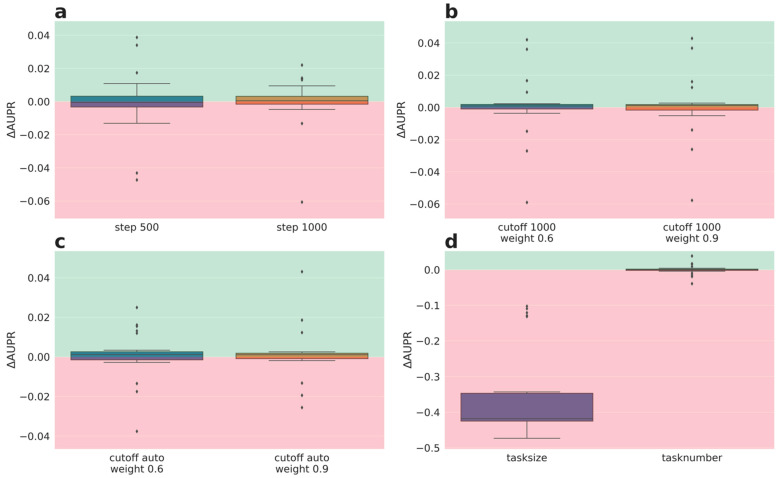
Phase II results of different weighting schemes averaged over 5 partners and 5 folds for lower quartile task (AUPR): (**a**) continuous weighting scheme with weight 0.02 and steps left: 1000 and right: 500, (**b**) fixed weighting scheme with cutoff 1000 and left: weight of 0.6 and right: weight of 0.9, (**c**) fixed weighting scheme with 95% quantile cutoff and left: weight 0.6 and right weight 0.9, (**d**) left: weighting based on task size, right: weight set to one divided by number of datapoints. Green: better performance than baseline (1), red: worse performance than baseline.

The performances of weighting schemes of phase II were analyzed in more detail. Therefore, the average synoptic performances were deconvoluted into the performances of the median and the lower quartile tasks. The delta performances for the lower quartile tasks (more informative tasks compared to the median tasks, and also more challenging tasks due to higher label imbalance) are depicted in Figure 2 (delta AUROC) and Figure 3 (delta AUPR). Full performance plots (including synoptic delta performances and delta performances of the median tasks) are given in the Supplementary Materials (Figures S1 and S2).

All but one (task size) weighting scheme improved the AUROC performance of the quartile task to a similar extent (see Figure 2). In contrast, for AUPR all tested weighting schemes except weighting wrt. task size resulted in a low delta AUPR close to zero (see Figure 3), and therefore were essentially equivalent in performance to the baseline (equal weight of 1). Weighting based on task size clearly performs worse than the baseline weighting (Figure 3d left). The computation of the weights based on task size leads to an extreme distribution with many very small weights and a weight that is overall (sum over all tasks) much smaller than the overall weight of the other weighting schemes.

An explanation for the small delta AUPR values could be that the AUPR metric is dependent on the fraction actives, which does not change through the different weighting schemes and thus masks the impact of the different weighting schemes. An analysis of the correlation of AUPR and AUROC values to several factors, e.g., fraction actives, can be found in the Supplementary Materials (Figure S3).

Subsequent to this analysis, we were curious whether other task characteristics besides task size, used to compute task weights, further improve predictive performance. Hence, in phase III other task characteristics like label balance and fraction actives were used to compute the task weights.

### 2.3. Phase III

#### 2.3.1. Synoptic Performance Analysis

Due to the small performance deltas in phases I–II, a significance test was used in phase III (see Table S3). Furthermore, in phase III weighting schemes based on the label balance of the tasks or the fraction of actives in the tasks were assessed. Two general settings can be distinguished in phase III: a global weighting strategy (inter assay) and a weighting strategy that remains within one assay (intra assay). For global weighting strategies, the label balance or fraction actives for the task at hand is compared to the overall (global) fraction actives/label balance, whereas for intra assay weighting strategies, the label balance or fraction actives of that task is only related to the label balance or fraction actives of other tasks originating from the same assay.

No task that performs significantly better than the baseline could be identified for any of the phase III weighting schemes (see Table S3). On the other hand, some of the weighting schemes have a considerably high percentage of tasks performing worse than the baseline. Particularly, up-weighting balanced tasks globally (“balance up weight”) leads on average over three partners and five folds to 74% of tasks performing statistically significantly worse than the baseline.

However, phase III also identified weighting schemes that result in only a very small percentage of significantly worse performing tasks (“fractive down weight”, “fractive up weight”, “intra down weight balanced” in Table S3). Especially for these weighting schemes, an analysis of the convergence speed is of interest to determine whether computational costs could be saved with one of these schemes while not decreasing performance (results see below, Section 2.3.3).

On a synoptic performance level, no weighting scheme of any of the three phases could be identified that performs considerably better for all participating partners than the baseline (1). However, the practical value of both tasks per assay (median and lower quartile) is not equal, and a deterioration of the median-based task performance may be acceptable if the lower quartile task performance improves. Therefore, the pure quartile-based task performance was analyzed for phase III weighting schemes.

#### 2.3.2. Quartile Task Performance

**Figure 4:**
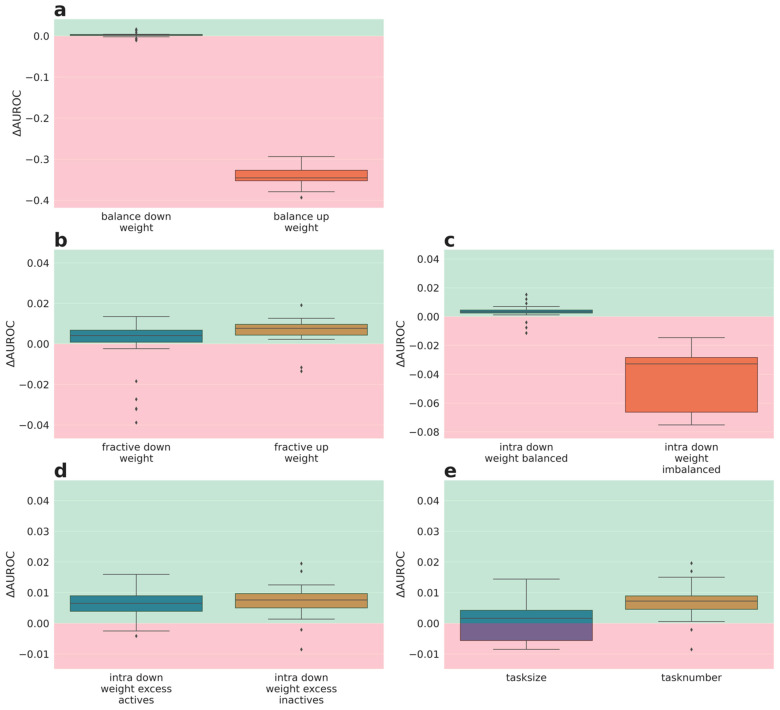
Phase III results averaged over 5 partners and 5 folds for lower quartile tasks performances (AUROC): (**a**) global weighting wrt. label balance, left: down-weighting balanced tasks, right: down-weight imbalanced tasks, (**b**) global weighting wrt. fraction actives, left: down-weighting excess of actives, right: down-weight excess of inactives, (**c**) intra assay weighting wrt. label balance, left: down-weighting balanced tasks, right: down-weight imbalanced tasks, (**d**) intra assay weighting wrt. fraction actives, left: down-weighting excess of actives, right: down-weight excess of inactives, (**e**) left: weight set wrt. number of datapoints, right: based on 1/task_number. Green: better performance than baseline (1), red: worse performance than baseline.

**Figure 5:**
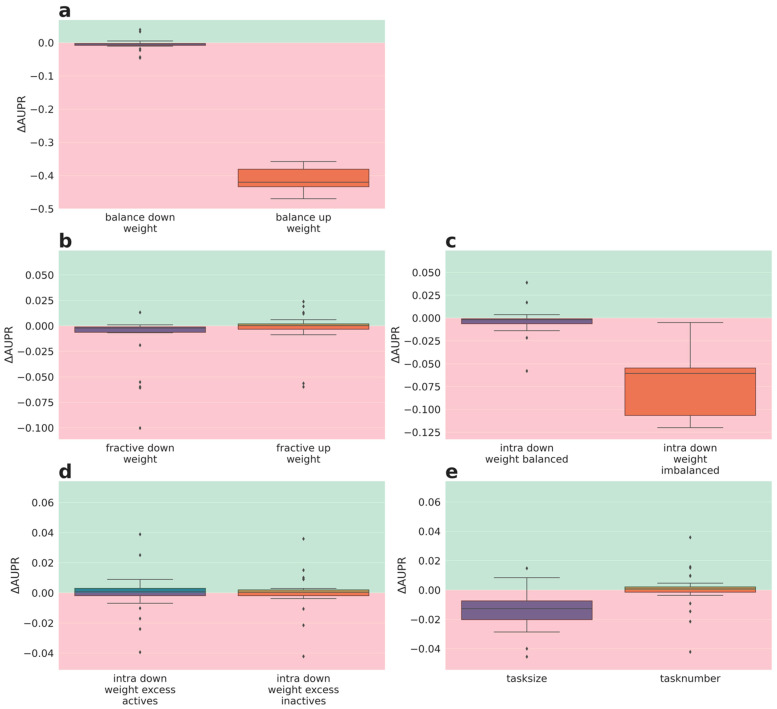
Phase III results averaged over 5 partners and 5 folds for lower quartile tasks performances (AUPR): (**a**) global weighting wrt. label balance, left: down-weighting balanced tasks, right: down-weight imbalanced tasks, (**b**) global weighting wrt. fraction actives, left: down-weighting excess of actives, right: down-weight excess of inactives, (**c**) intra assay weighting wrt. label balance, left: down-weighting balanced tasks, right: down-weight imbalanced tasks, (**d**) intra assay weighting wrt. fraction actives, left: down-weighting excess of actives, right: down-weight excess of inactives, (**e**) left: weight set wrt. number of datapoints, right: based on 1/task_number. Green: better performance than baseline (1), red: worse performance than baseline.

The performances of weighting schemes of phase III were analyzed in more detail, as for phase 2. The delta performances for the lower quartile tasks are depicted in Figure 4 (delta AUROC) and Figure 5 (delta AUPR). Full performance plots (including synoptic delta performances and delta performances of the median tasks) are given in the Supplementary Materials (Figures S4 and S5).

Interestingly, the delta AUROC performance evaluation (Figure 4) reveals that weighting based on the fraction actives and number of sub-tasks (Figure 4b,d,e, respectively) is superior to the baseline weighing (1) for the quartile tasks. Surprisingly, this positive effect is independent from whether tasks with a high fraction of actives got down- or up-weighted. Down-weighting imbalanced tasks is again detrimental (orange boxes Figure 4a,c). Down-weighting balanced tasks (purple boxes Figure 4a,c) and weighting based on task size (Figure 4e) had no strong impact on delta AUROC performance.

**Figure 6:**
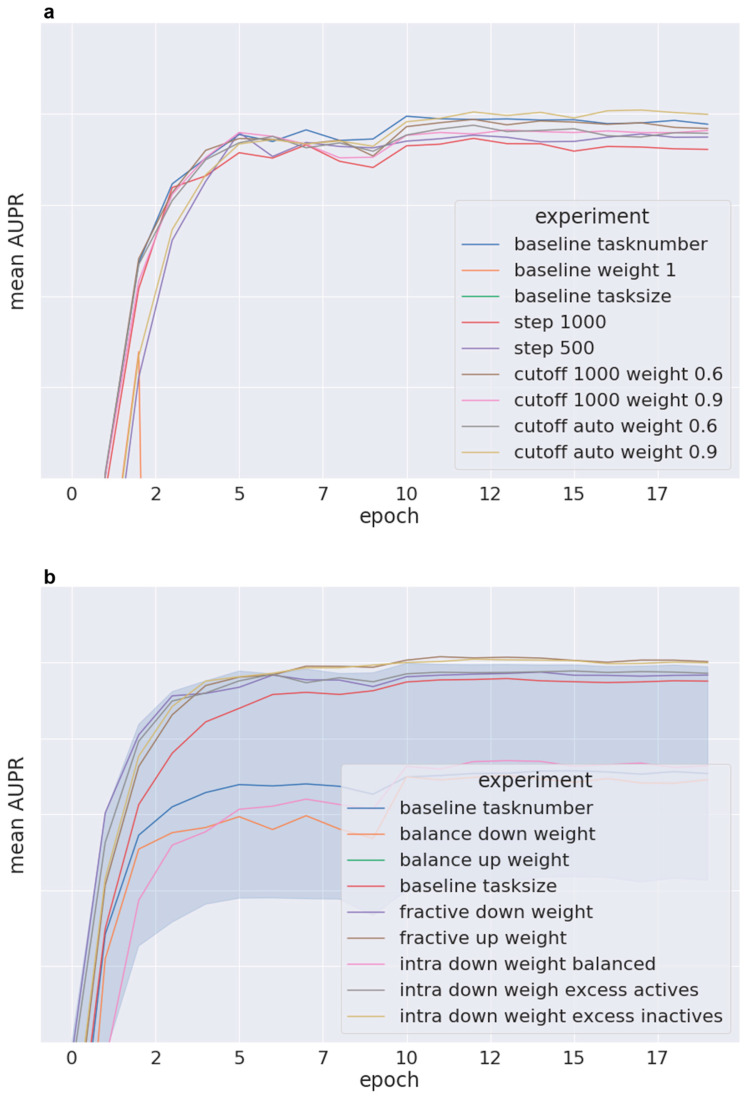
Convergence results averaged over 5 folds for synoptic performance (median and lower quartile, AUPR): (**a**) phase II weighting schemes, (**b**) phase III weighting schemes.

Nevertheless, the AUPR performance evaluation (Figure 5), confirms the results from the synoptic performance analysis (see above, Section 2.3.1) and shows only a weak impact of the weighting schemes of phase III on the performance of the quartile tasks (small delta AUPR values). Again, the weighting scheme that down-weights imbalanced tasks (orange boxes Figure 5a and Figure 6c) is an exception with a negative impact on performance. Moreover, weighting based on task size (Figure 5e) also performs worse; the latter observation being contradictory to the delta AUROC performances.

The performance evaluation based on the lower quartile tasks demonstrated phase III weighting schemes with an improved AUROC performance compared to the baseline. Interestingly, most of these beneficial weighting schemes are based on the fraction of actives. Remarkably, both directions, i.e., down-weighting an excess of actives or inactives, lead to superior performance. On the other hand, an opposite trend was observed for weighting based on balance. Here, down-weighting balanced task is clearly superior to down-weighting imbalanced tasks. Together with the results achieved for down-weighting according to the fraction of actives, one can conclude that the positive performance delta rather originates from up-weighting tasks with either high or low fraction of actives than from down-weighting the opposite, because down-weighting both (down-weighting imbalanced) leads to a much worse performance than up-weighting imbalanced tasks. Noteworthily, the observations made by the intra-assay weighting schemes can be transferred to the global weighting schemes. In contrast to the AUROC-based performance analysis, no weighting scheme of phase III demonstrated a strong positive delta performance compared to the baseline (1) wrt. AUPR. However, preserving performance while reducing learning time would also be favorable. Thus, the impact of different weighting schemes on the speed of convergence was assessed.

#### 2.3.3. Speed of Convergence

The speed of convergence was assessed for weighting schemes of phase II and phase III (see Figure 6a,b, respectively). Those schemes performing better than the baseline (1) reached the plateau of the learning curve at a similar number of epochs as the baseline (see Figure 6).

None of the weighting schemes with reasonable performance exhibit accelerated learning.

## 3. Materials and Methods

### 3.1. Data Preparation

Data was prepared in a standardized way throughout all pharmaceutical companies involved in this work utilizing MELLODDY-TUNER [https://github.com/melloddy/MELLODDY-TUNER, accessed on 10 November 2021]. In total six pharma companies (Amgen, Bayer, Boehringer Ingelheim, GSK, Janssen Pharmaceutica NV, Novartis) performed the experiments of this study. Each of the six data sets contained 100,000–2,000,000 unique compounds and 3000–26,000 tasks. The chemical space was analyzed for one private data set and shows that the majority of compounds are unsurprisingly in a drug-like chemical space with a median clogP of ~2.9 and a median molecular mass of ~390. This is in good alignment with other analyses [17], and in general other private pharmaceutical data sets are expected to populate a similar chemical space regarding physico-chemical properties [18]. In addition to the weighting performed by MELLODDY-TUNER, further weighting schemes were investigated. The weighting schemes applied can be divided into four categories:

1. Baseline.
2. Fixed weighing schemes.
3. Continuous weighting schemes.
4. Weighting based on task properties (here task size, fraction actives or class label balance) and number of sub-tasks.

### 3.2. Baseline

The baseline used in this study is a weight of 1 for each task resulting in an equal weight for all tasks. In the performance plots, delta performance compared to this baseline is depicted if not stated otherwise.

### 3.3. Fixed Weighting Schemes

In the fixed scheme, all tasks with more than a certain number of datapoints (cutoff) were down-weighted to a fixed value which is smaller than one. Tasks below the cutoff obtain a weight of 1/task_number. In this work, the cutoff was 1000 datapoints or the 95% quantile of the task size. Weights studied were: 0.1, 0.25, 0.5, 0.6, 0.75 and 0.9. The weights were divided by the number of sub-tasks. Further cutoffs and down-weighting values were studied in a pretest (see Supplementary Materials Table S1) but were not selected for analysis by all partners due to lower performance.

### 3.4. Continuous Weighting Schemes

In the continuous scheme, all tasks with more than a certain number of datapoints (cutoff) were down-weighted by a value which increased by the amount the task’s data size exceeded the cutoff. Tasks below the cutoff obtained a weight of 1/task_number. In this work, two cutoffs (500 and 1000) were investigated. Every 500 respective 1000 datapoints the weight of the corresponding task was reduced by 0.02, e.g., if a task had 3600 datapoints, the weight is in the 500 cutoff scheme 1 − (3600/500) × 0.02 = 1 − 0.144 = 0.856, and in the 1000 cutoff scheme 1 − (3600/1000) × 0.02 = 1 − 0.072 = 0.928 (assuming only one task per assay). Further cutoffs and down-weighting values were studied in a pretest (see Supplementary Materials Table S1), but were not selected for analysis by all partners due to lower performance.

### 3.5. Weighting Based on Number of Sub-Tasks and Task Size

Based on the task size two schemes were analyzed. The calculation of the weighting schemes based on task number and task size is given below:

let D⊆A×ℝ×{=, 〈, 〉}

*be the data set used, where A is the set of assays*

$$with\;D_{i}\subseteq D$$

*the data corresponding to assay a_i_*

$$and\;D_{ij}\subseteq D_{i}$$

*the data corresponding to the jth task of assay* $a_{i}$

$$then\;let\;t_{ij}:\;D_{ij}\to \left\{0,1\right\}$$

*be the corresponding task,*

$$t_{ij}\in \;T_{i}\subseteq T$$

*with T_i_ the tasks corresponding to assay* $a_{i}$*, and*  T *the set of all tasks*

*then the first weighting strategy is*

$$w_{ij}^{basic}=\frac{1}{number\;of\;tasks\;for\;the\;assay}=\frac{1}{\left|T_{i}\right|}$$

*The next takes data volumes into consideration*

$$w_{ij}^{volume}=\frac{1}{number\;of\;datapoints\;for\;task\;j,\;assay\;i}=\frac{1}{\left|D_{ij}\right|}$$

*Finally, a scaled version of this that leaves the average task weight invariantly one*

$$w_{ij}^{avg}=\frac{number\;of\;tasks}{\sum_{t\;\epsilon \;T}\frac{1}{number\;of\;datapoints\;of\;task\;t}}\;*\;\frac{1}{number\;of\;datapoints\;for\;the\;task\;ij}=\frac{\left|T\right|}{\sum_{t_{kl}\epsilon \;T}{\left|D_{kl}\right|}^{-1}}*w_{ij}^{volume}$$

### 3.6. Weighting Based on Fraction Actives or Class Label Balance

The fraction actives as well as the label balance are factors influencing the difficulty of the classification task. Some studies suggest that better predictive performance can be achieved when weighting either difficult tasks higher (giving them a higher priority) [11] or down-weighting them (giving a lower priority) [12,19]. The latter is probably especially the case if the data is noisy [10]. Thus, both directions were tested within this study. In addition, to giving easy tasks a higher priority the down-weighting of tasks with a low fraction of actives can be seen as an up-sampling of the active’s class, which is usually underrepresented in drug discovery related tasks. Moreover, two different schemes of weighting are explored. On the one hand, the weight is only calculated based on the fraction actives respective label balance within the tasks corresponding to the same assay, leading to a weight of one in sum over the tasks in that assay (intra-assay). Thus, this scheme sees the sums of weights over one assay held at one, with label balance respective fraction actives only adjusted for between tasks on that assay. On the other hand, the weights are calculated considering all tasks’ fraction actives respective label balance (inter-assay). The weights were calculated as follows:

Here, fraction active means:

$$f_{ij}^{active}=\frac{\left|\left\{\left(x,mod\right)\;\epsilon \;D_{ij}\;\;\;s.t.\;\;\;\;t_{ij}\left(\left(x,mod\right)\right)=1\right\}\right|}{\left|D_{ij}\right|}$$

#### 3.6.1. Intra-Assay

based on fraction actives:

down-weight excess of inactives:

$$w_{ij}^{inactives↓}=\frac{f_{ij}^{active}}{\sum_{t_{ik}\;\epsilon \;T_{i}}f_{ik}^{active}}$$

down-weight excess of actives (normalized inverse of above weight):

$$w_{ij}^{actives↓}={\left(w_{ij}^{inactives↓}\right)}^{-1}*\frac{1}{\sum_{t_{ik}\;\epsilon \;T_{i}}\;{\left(w_{ij}^{inactives↓}\right)}^{-1}}$$

based on label balance

down-weight imbalanced (more extreme fractions penalized, then weight normalized):

$$w_{ij}^{imbalanced↓}=\frac{1}{\left|0.5-f_{ij}^{active}\;\right|}*{\left(\underset{t_{ij}\;\epsilon \;T_{i}}{\sum }\frac{1}{\left|0.5-f_{ij}^{active}\;\right|}\right)}^{-1}$$

down-weight balanced (extreme fractions favored, then normalized):

$$w_{ij}^{balanced↓}=\left|0.5-f_{ij}^{active}\;\right|*{\left(\underset{t_{ij}\;\epsilon \;T_{i}}{\sum }\left|0.5-f_{ij}^{active}\;\right|\right)}^{-1}$$

**Figure 7:**
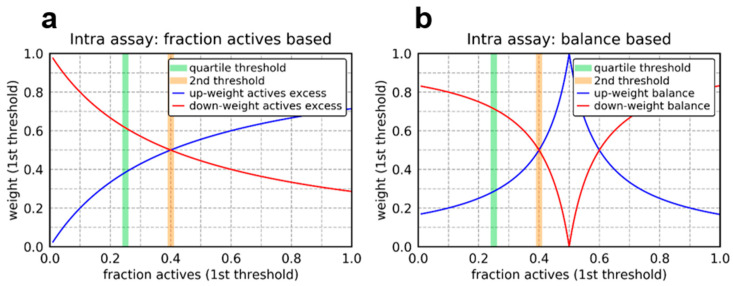
Visualization of intra assay weighting functions for the first threshold assuming a fraction of actives of 0.4 for the second threshold: (**a**) fraction actives based weighting and (**b**) balance based weighting.

The intra-assay weighting functions are depicted in Figure 7.

#### 3.6.2. Inter Assay

based on fraction actives:

down-weight excess of inactives

$$w_{ij}^{inactives↓}=\frac{f_{ij}^{active}*\left|A\right|}{\sum_{t_{kl}\;\epsilon \;T}f_{kl}^{active}}$$

down-weight excess of actives

$$s_{i}=\underset{t_{ij}\;\epsilon \;T_{i}}{\sum }f_{ij}^{active}$$

$$w_{ij}^{actives↓}=\frac{1}{f_{ij}^{active}}*\;\frac{1}{s_{i}*\sum_{t_{ik}\;\epsilon \;T_{i}}{\left(f_{ik}^{active}\right)}^{-1}}*\frac{\left|A\right|}{\sum_{a_{k}\epsilon A}\left(\left|T_{k}\right|{\left(s_{k}\right)}^{-1}\right)}$$

based on label balance

down-weight imbalanced

$$w_{i}=\frac{0.5*\left|A_{i}\right|}{\left|0.5-fraction\;actives\;of\;i\right|}*\frac{\frac{0.5*\left|T\right|}{\left|0.5*\right|A_{i}\left|-\sum_{t\;\epsilon \;A_{i}}fraction\;actives\;of\;t\right|}*\frac{\left|A\right|}{\sum_{s\;\epsilon \;A_{i}}\frac{0.5*\left|T\right|}{\left|0.5*\right|A_{s}\left|-\sum_{t\;\epsilon \;A_{s}}fraction\;actives\;of\;t\right|}}}{\sum_{t\;\epsilon \;A_{i}}\frac{0.5*\left|A_{i}\right|}{\left|0.5-fraction\;actives\;of\;t\right|}}\;w_{ij}^{imbalanced↓}=\frac{0.5*\left|T_{i}\right|}{\left|0.5-f_{ij}^{active}\;\right|}*\frac{\frac{0.5*\left|T\right|}{\left|\sum_{t_{ik}\;\epsilon \;T_{i}}0.5-f_{ik}^{active}\;\right|}*\frac{\left|A\right|}{0.5*\left|T\right|*\left|T_{i}\right|{\left|\sum_{t_{ik}\;\epsilon \;T_{i}}0.5-f_{ik}^{active}\right|}^{-1}}}{0.5*\left|T_{i}\right|*\sum_{t_{ik}\;\epsilon \;T_{i}}{\left|0.5-f_{ik}^{active}\right|}^{-1}\;}$$

$$w_{ij}^{imbalanced↓}=\frac{0.5*\left|T_{i}\right|}{\left|0.5-f_{ij}^{active}\;\right|}*\frac{\frac{\left|A\right|}{\left|T_{i}\right|}}{0.5*\left|T_{i}\right|*\sum_{t_{ik}\;\epsilon \;T_{i}}{\left|0.5-f_{ik}^{active}\right|}^{-1}\;}$$

$$w_{ij}^{imbalanced↓}=\frac{1}{\left|0.5-f_{ij}^{active}\;\right|}*\frac{\left|A\right|}{\left|T_{i}\right|*\sum_{t_{ik}\;\epsilon \;T_{i}}{\left|0.5-f_{ik}^{active}\right|}^{-1}\;}$$

down-weight balanced

$$w_{i}=\frac{\left|0.5-fraction\;actives\;of\;i\right|}{0.5*\left|A_{i}\right|}*\frac{\frac{\left|0.5*\right|A_{i}\left|-\sum_{t\;\epsilon \;A_{i}}fraction\;actives\;of\;t\right|\;}{0.5*\left|T\right|}*\frac{\left|A\right|}{\sum_{s\;\epsilon \;A_{i}}\frac{\left|0.5*\left|A_{s}\right|-\sum_{t\;\epsilon \;A_{s}}fraction\;actives\;of\;t\right|}{0.5*\left|T\right|}}}{\sum_{t\;\epsilon \;A_{i}}\frac{\left|0.5-fraction\;actives\;of\;t\right|}{0.5*\left|A_{i}\right|}}$$

$$w_{ij}^{balanced↓}=\frac{\left|0.5-f_{ij}^{active}\right|}{0.5*\left|T_{i}\right|}*\frac{\frac{\left|\sum_{t_{ik}\;\epsilon \;T_{i}}0.5-f_{ik}^{active}\;\right|}{0.5*\left|T\right|}*\frac{\left|A\right|}{\frac{\left|T_{i}\right|*\left|\sum_{t_{ik}\;\epsilon \;T_{i}}0.5-f_{ik}^{active}\;\right|}{0.5*\left|T\right|}}}{\frac{1}{0.5*\left|T_{i}\right|}*\sum_{t_{ik}\;\epsilon \;T_{i}}\left|0.5-f_{ik}^{active}\right|\;}$$

$$w_{ij}^{balanced↓}=\frac{\left|0.5-f_{ij}^{active}\right|}{0.5*\left|T_{i}\right|}*\frac{\frac{\left|A\right|}{\left|T_{i}\right|}}{\frac{1}{0.5*\left|T_{i}\right|}*\sum_{t_{ik}\;\epsilon \;T_{i}}\left|0.5-f_{ik}^{active}\right|\;}$$

$$w_{ij}^{balanced↓}=\left|0.5-f_{ij}^{active}\right|*\frac{\left|A\right|}{\left|T_{i}\right|*\sum_{t_{ik}\;\epsilon \;T_{i}}\left|0.5-f_{ik}^{active}\right|\;}$$

### 3.7. Training

The prepared data were subsequently used to train a feedforward neural network using SparseChem [https://github.com/melloddy/SparseChem, accessed on 10 November 2021]. SparseChem is a package for machine learning models for biochemical applications capable of high-dimensional sparse input. The data were split into five folds (subsets) using locality sensitive hashing on molecular fingerprint features [20]. Three folds were used for training, whereas one was used as test and the other as validation fold. The hyperparameters as named in SparseChem, which were tested using five folds and cross validation, are given in Table 2.

The results from the best hyperparameter set were used for subsequent evaluations and analyses.

### 3.8. Evaluation

The results were evaluated based on the following criteria: difference in predictive performance (between baseline performance and performance of weighting scheme under investigation) measured as area under the precision-recall or receiver operating characteristics curve (AUPR, respectively AUROC), the number of statistically significant better or worse tasks based on AUROC, and the convergence speed. Convergence speed was determined as a plot of performance against epoch number. AUPR and AUROC were averaged over all tasks fulfilling the criteria, i.e., minimal number of actives and inactives and depending on the analysis, e.g., only tasks of the lower quartile threshold.

To estimate whether two tasks had statistically significant different AUROC values, a *p*-value was calculated based on a Z-test comparing the two AUROC values. It has been shown [21] that the relatedness between the AUROC and the Wilcoxon statistic allows for obtaining an expression for the standard deviation of the AUROC:

$$s\left(AUC\right)=sqrt\;\frac{AUC\left(1-AUC\right)+\left(n1-1\right)\left(Q1-AUC^{2}\right)+\left(n0-1\right)\left(Q2-AUC^{2}\right)}{n1*n0}$$

with

$$Q1=\frac{AUC}{2-AUC}$$

$$Q2=\frac{A2*AUC^{2}}{1+AUC}$$

and *n*1 and *n*0 are the number of actives and inactives, respectively, in the fold used for evaluation. Intuitively, for this binary classification setting, both the nonparametric Wilcoxon statistic and the AUROC are measures for the quality of ranking actives versus inactive samples, and depend fully on the rank between the samples.

Once the standard deviations of two AUROC values (*sd*1 and *sd*2) have been calculated, assuming normality, a Z-test can be used to compare the difference of the mean AUROC values:

$$Z=\frac{AUC1-AUC2}{sqrt\;(\left(sd1^{2}+sd2^{2}\right))}$$

Z being the z-score from which a *p*-value can be calculated using the cumulative distribution function of a normal distribution with mean 0, up to Z. If 1-(*p*-value) exceeds the confidence level of 95%, the null hypothesis of the two AUROC values being statistically equal is rejected.

## 4. Conclusions

Weighting tasks in multi-task models is a current area of considerable scientific interest. In this article, we analyzed several weighting schemes in the context of federated multi-task learning on pharmaceutical industry data in a privacy preserving setting, which was realized for the first time at this large a scale in drug discovery. In general, our models were resilient to most perturbations to the weights, indicating that the limiting factor on performance is the underlying information in the data and the model architecture. The weighting schemes that lead to a drop in performance (down-weight imbalanced and weighting based on task size) often would have led to extreme distributions of weights due to their reciprocal style of computation. Thus, a few very small x values would have dominated the total contribution to sums used for normalization. All analyzed weighting schemes with comparable AUPR performance also have similar speeds of convergence. Flexible and continuous weighting schemes, as well as weighting based on the fraction of actives (fractive up weight and intra assay weighting based on fraction actives), and based on the number of sub-tasks were shown to be beneficial both regarding synoptic and lower quartile task AUROC performance. Noteworthily, this result is consistent through six different pharmaceutical industry data sets. Weighting based on the number of tasks is furthermore especially suited to federated learning, because it prevents partners from artificially increasing their weight by adding more tasks.

## Figures and Tables

**Table 1:** Overview of analyzed weighting schemes. Experiments were performed in three phases blue: I, yellow: II, green: III. “Cutoff” determines the task size threshold above which down-weighting according to weights was applied either only once (fixed scheme) or as often as “cutoff” fit into the task size (continuous scheme).

	Fixed	Continuous	Baseline
**cutoff**	1000	-	based on task number
**weights**	0.1, 0.25, 0.5, 0.75		
**cutoff**	1000, 95% quantile	500, 1000	-
**weights**	0.6, 0.9	0.02	1
**weights**	-	wrt. fraction actives, wrt, label balance	1

**Table 2:** Overview of analyzed hyperparameters. Two numbers, e.g., “1200 1200”, in the “hidden_size” row indicate two hidden layers. “middle_dropout” only applies if the network consists of multiple layers.

Hyperparameter	Values					
hidden_sizes	800	1200	1600	2000	1200 1200	1600 1600
Number of hidden layers	1	1	1	1	2	2
last_dropout	0.4					
middle_dropout	0.4					
min_samples_auc	50					
weight_decay	1 × 10^−5^					
epochs	20					
lr_steps	10					

## Data Availability

The code developed within the MELLODDY project and applied in this work is available under https://github.com/melloddy/SparseChem and https://github.com/melloddy/MELLODDY-TUNER.

## Supplementary Materials

The following are available online, Figure S1: Full performance plots phase II AUROC, Figure S2: Full performance plots phase II AUPR, Figure S3: Correlation analysis of AUPR and AUROC, Table S1: Tested weighting schemes during a pretest for phase II, Figure S4: Full performance plots phase III AUROC, Figure S5: Full performance plots phase III AUPR.

## Sample Availability

Samples are not available from the authors.
