# Supplementary material for: Don’t Overweight Weights: Evaluation of Weighting Strategies for Multi-Task Bioactivity Classification Models
Source: Molecules. 2021 Nov 18;26(22):6959. doi: 10.3390/molecules26226959 (PMC8620420; doi:10.3390/molecules26226959)
Supplement: Supplementary file 1 [file molecules-26-06959-s001.zip › molecules-1451635-supplementary.pdf]

molecules

Supporting Material

Don’t overweight weights: Evaluation of weighting strategies for multi-task bioactivity classification models

Lina Humbeck[1*], Tobias Morawietz[2], Noe Sturm[3], Adam Zalewski[4], Simon Harnqvist[5§], Wouter Heyndrickx[6], Matthew Holmes[5], Bernd Beck[1]

[1] Medicinal Chemistry Department, Boehringer Ingelheim Pharma GmbH & Co. KG, Birkendorfer Str. 65, 88397, Biberach an der Riss, Germany; lina.humbeck@boehringer-ingelheim.com and bernd.beck@boehringer-ingelheim.com
[2] Bayer AG, Pharmaceuticals, R&D, Digital Technologies, Computational Molecular Design, 42096 Wuppertal, Germany; tobias.morawietz@bayer.com
[3] Novartis Institutes for BioMedical Research, CH-4002 Basel, Switzerl; noe.sturm@novartis.com
[4] Amgen Research (Munich) GmbH, Staffelseestraße 2, 81477 Munich, Germany; azalewsk@amgen.com
[5] Computational Sciences, GlaxoSmithKline, Gunnels Wood Road, Stevenage SG1 2NY, United Kingdom; seh589@york.ac.uk and mwh35@bath.ac.uk
[6] Janssen Pharmaceutica N.V., Turnhoutseweg 30, 2340 Beerse, Belgium; wheyndri@its.jnj.com
* Correspondence: lina.humbeck@boehringer-ingelheim.com
§ Current address: Department of Biology, University of York, YO10 5DD, York UK

Table S1. Tested weighting schemes during a pretest for phase II. Bold: selected parameters for testing by multiple partners in phase II.

	fixed	continuous	baseline
**thresholds**	**1000**, 2000, 4000, 5000, **95% quantile**	100, **500**, **1000**, 5000, 95% quantile	-
**weights**	**0.6**, 0.7, 0.8, **0.9**	0.01, **0.02**, 0.04, 0.05, 0.08, 0.1	**1**

Table S2. Phase III results of different weighting schemes averaged over 3 partners and 5 folds for synoptic performance (median and lower quartile task, AUROC) compared to baseline (1) performance. Fractive: fraction of actives, *statistically significant.

	**% better tasks* (averaged over 3 partner)**	**% worse tasks* (averaged over 3 partner)**
Balance down weight	0	2.36
Balance up weight	0	74.08
Based on task size	0	2.24
Fractive down weight	0	0.19
Fractive up weight	0	0.48
Intra down weight balanced	0	1.36
Intra down weigh excess actives	0	1.65
Intra down weight excess inactives	0	2.95
Intra down weight imbalanced	0	5.70
Based on task number	0	5.70

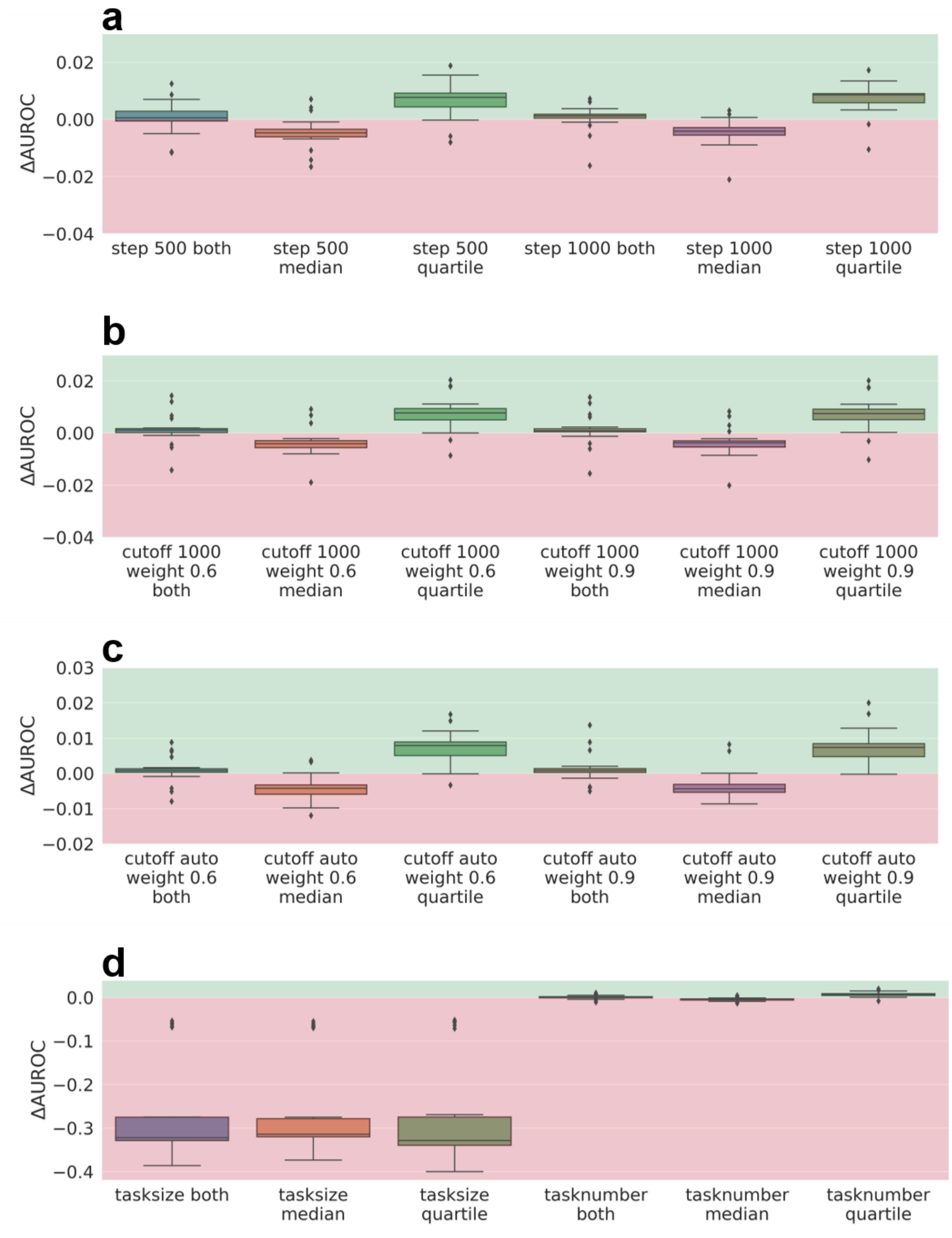
a
ΔAUROC
0.02
0.00
−0.02
−0.04
step 500 both
step 500 median
step 500 quartile
step 1000 both
step 1000 median
step 1000 quartile
b
ΔAUROC
0.02
0.00
−0.02
−0.04
cutoff 1000 weight 0.6 both
cutoff 1000 weight 0.6 median
cutoff 1000 weight 0.6 quartile
cutoff 1000 weight 0.9 both
cutoff 1000 weight 0.9 median
cutoff 1000 weight 0.9 quartile
c
ΔAUROC
0.03
0.02
0.01
0.00
−0.01
−0.02
cutoff auto weight 0.6 both
cutoff auto weight 0.6 median
cutoff auto weight 0.6 quartile
cutoff auto weight 0.9 both
cutoff auto weight 0.9 median
cutoff auto weight 0.9 quartile
d
ΔAUROC
0.0
−0.1
−0.2
−0.3
−0.4
tasksize both
tasksize median
tasksize quartile
tasknumber both
tasknumber median
tasknumber quartile

Figure S1. Phase II results of different weighting schemes averaged over 5 partners and 5 folds for synoptic and deconvoluted performances(median and lower quartile (blue and red boxes), only median (orange and purple boxes) and only lower quartile task (green and brown boxes), AUROC): (a) continuous weighting scheme with weight 0.02 and steps left: 500 and right: 1000, (b) fixed weighting scheme with cutoff 1000 and left: weight of 0.6 and right weight of 0.9, (c) fixed weighting scheme with 95% quantile cutoff and left: weight 0.6 and right weight 0.9, (d) left weighting based on task size, right: weight set to one divided by number of datapoints. Green: better performance than baseline (1), red: worse performance than baseline.

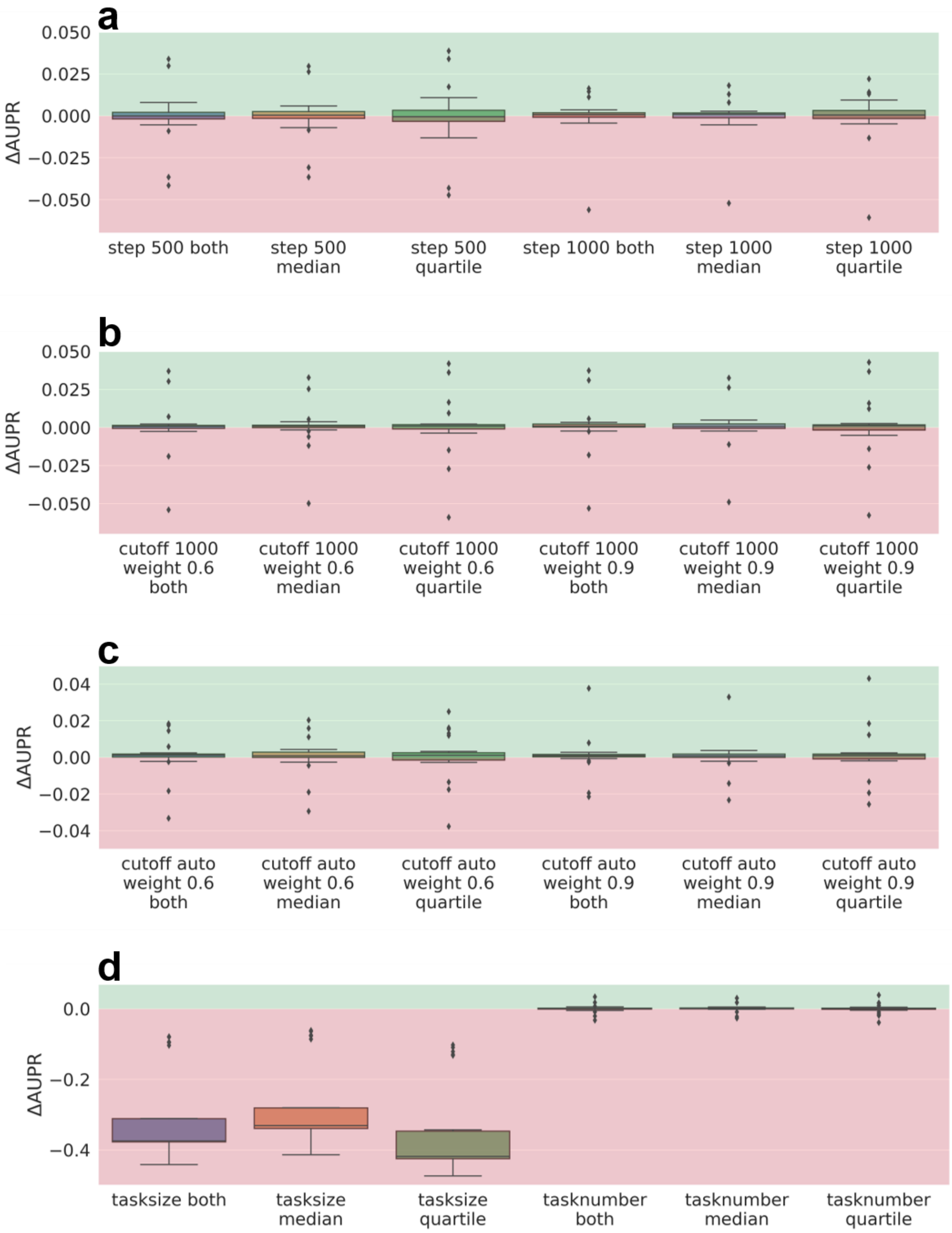
a
0.050
0.025
0.000
−0.025
−0.050
ΔAUPR
step 500 both
step 500 median
step 500 quartile
step 1000 both
step 1000 median
step 1000 quartile
b
ΔAUPR
cutoff 1000 weight 0.6 both
cutoff 1000 weight 0.6 median
cutoff 1000 weight 0.6 quartile
cutoff 1000 weight 0.9 both
cutoff 1000 weight 0.9 median
cutoff 1000 weight 0.9 quartile
c
0.04
0.02
0.00
−0.02
−0.04
ΔAUPR
cutoff auto weight 0.6 both
cutoff auto weight 0.6 median
cutoff auto weight 0.6 quartile
cutoff auto weight 0.9 both
cutoff auto weight 0.9 median
cutoff auto weight 0.9 quartile
d
0.0
−0.2
−0.4
ΔAUPR
tasksize both
tasksize median
tasksize quartile
tasknumber both
tasknumber median
tasknumber quartile

Figure S2. Phase II results of different weighting schemes averaged over 5 partners and 5 folds for synoptic and deconvoluted performances(median and lower quartile (blue and red boxes), only median (orange and purple boxes) and only lower quartile task (green and brown boxes), AUPR): (a) continuous weighting scheme with weight 0.02 and steps left: 500 and right: 1000, (b) fixed weighting scheme with cutoff 1000 and left: weight of 0.6 and right weight of 0.9, (c) fixed weighting scheme with 95% quantile cutoff and left: weight 0.6 and right weight 0.9, (d) left weighting based on task size, right: weight set to one divided by number of datapoints. Green: better performance than baseline (1), red: worse performance than baseline.

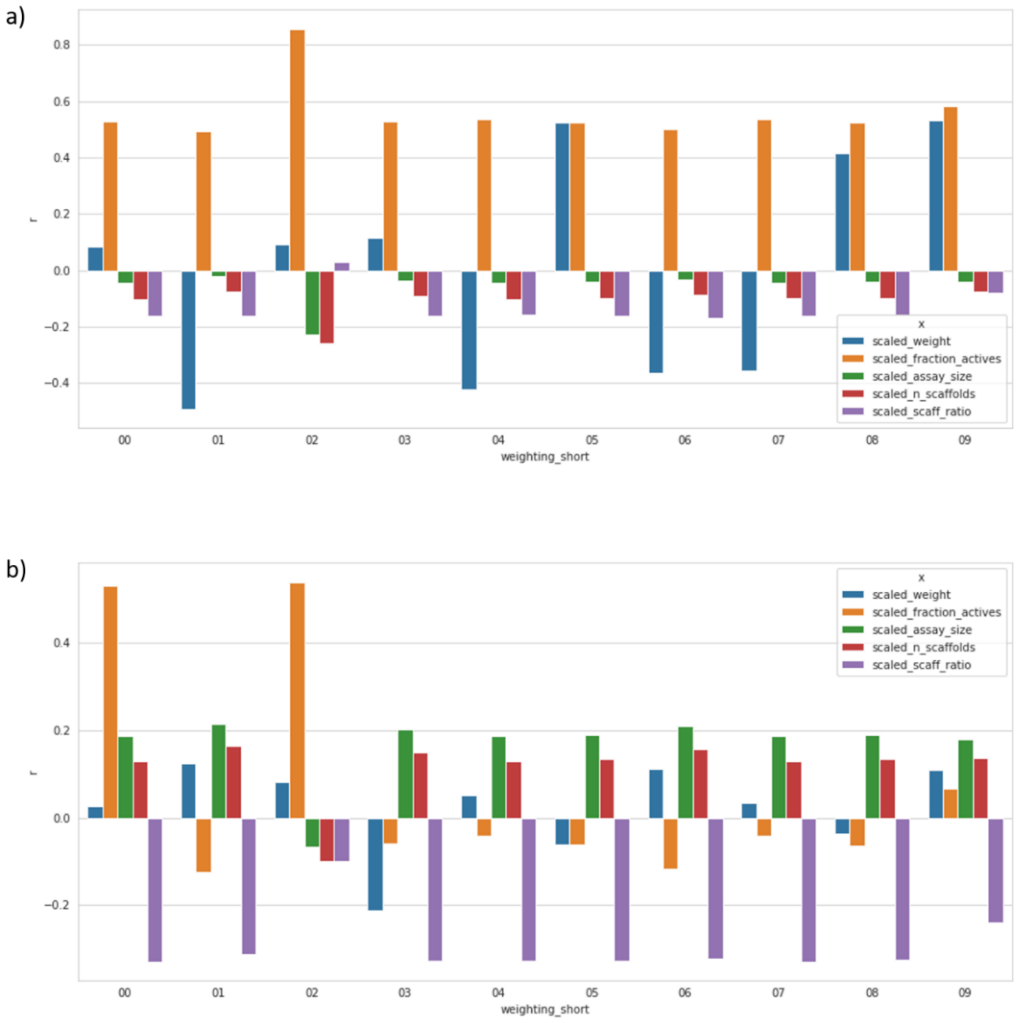

Figure S3. Correlation analysis for one partner of a) AUCPR and b) AUCROC. Results from a second partner are available and comparable and thus not shown. Analyzed factors are: blue: scaled weight, yellow: scaled fraction actives, green: scaled assay size, red: scaled number of scaffolds, purple: scaled scaffold ratio. 00: 1/task_number, 01: balance down weight, 02: balance up weight, 03: task size (phase III), 04: fractive down weight, 05: fractive up weight, 06: intra down weight balanced, 07: intra down weight excess actives, 08: intra down weight excess inactives, 09: intra down weight imbalanced.

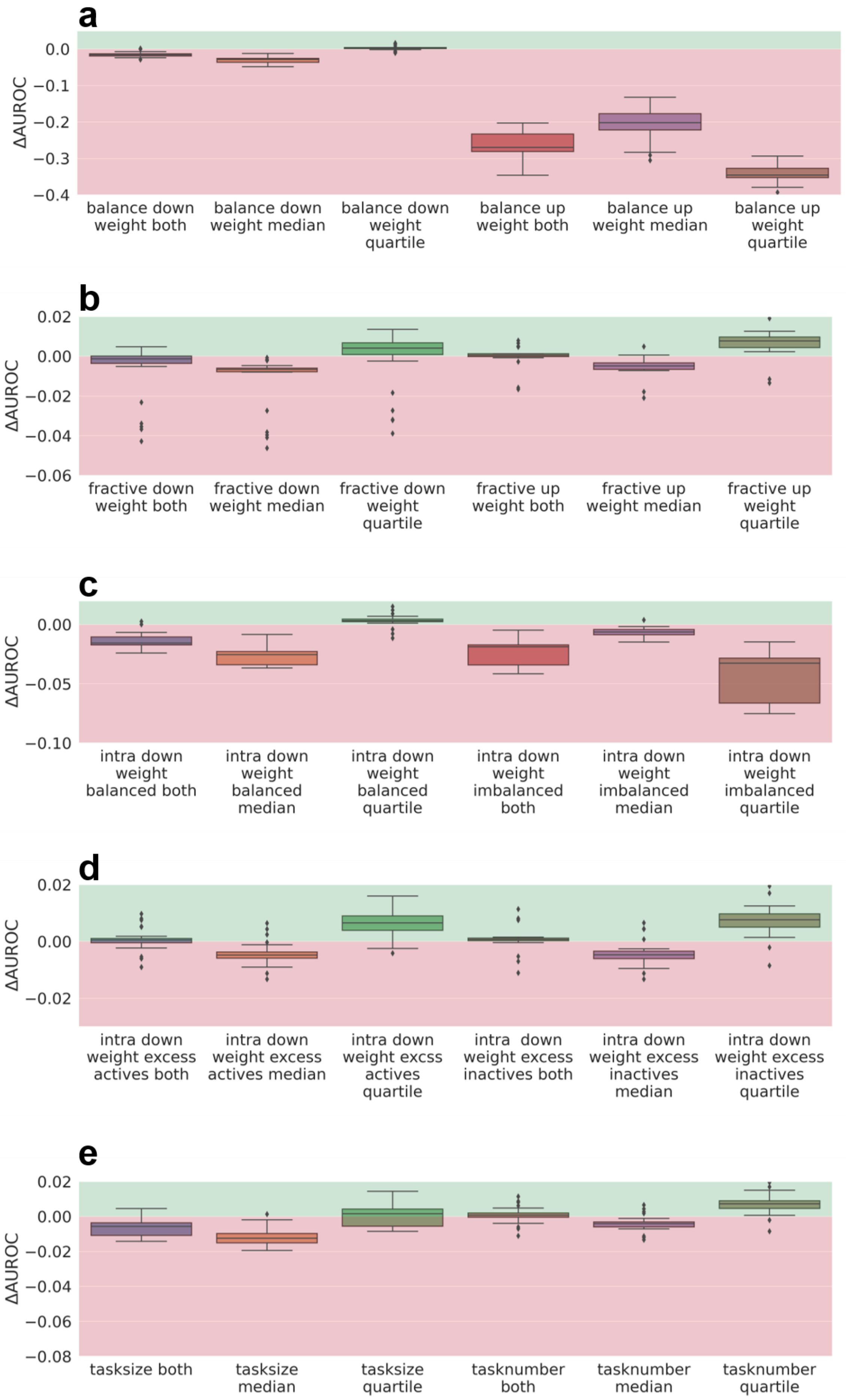
a
ΔAUROC
0.0
−0.1
−0.2
−0.3
−0.4
balance down weight both
balance down weight median
balance down weight quartile
balance up weight both
balance up weight median
balance up weight quartile
b
ΔAUROC
0.02
0.00
−0.02
−0.04
−0.06
fractive down weight both
fractive down weight median
fractive down weight quartile
fractive up weight both
fractive up weight median
fractive up weight quartile
c
ΔAUROC
0.00
−0.05
−0.10
intra down weight balanced both
intra down weight balanced median
intra down weight balanced quartile
intra down weight imbalanced both
intra down weight imbalanced median
intra down weight imbalanced quartile
d
ΔAUROC
0.02
0.00
−0.02
intra down weight excess actives both
intra down weight excess actives median
intra down weight excss actives quartile
intra down weight excess inactives both
intra down weight excess inactives median
intra down weight excess inactives quartile
e
ΔAUROC
0.02
0.00
−0.02
−0.04
−0.06
−0.08
tasksize both
tasksize median
tasksize quartile
tasknumber both
tasknumber median
tasknumber quartile

Figure S4. Phase III results averaged over 5 partners and 5 folds for synoptic and deconvoluted performances(median and lower quartile (blue and red boxes), only median (orange and purple boxes) and only lower quartile task (green and brown boxes), AUROC): (a) global weighting wrt. label balance left: down-weighting balanced tasks and right: down-weight imbalanced tasks, (b) global weighting wrt. fraction actives left: down-weighting excess of actives and right: down-weight excess of inactives, (c) intra assay weighting wrt. label balance left: down-weighting balanced tasks and right: down-weight imbalanced tasks, (d) intra assay weighting wrt. fraction actives left: down-weighting excess of actives and right: down-weight excess of inactives, (e) left: weight set wrt. number of datapoints and right: based on 1/task_number. Green: better performance than baseline (1), red: worse performance than baseline.

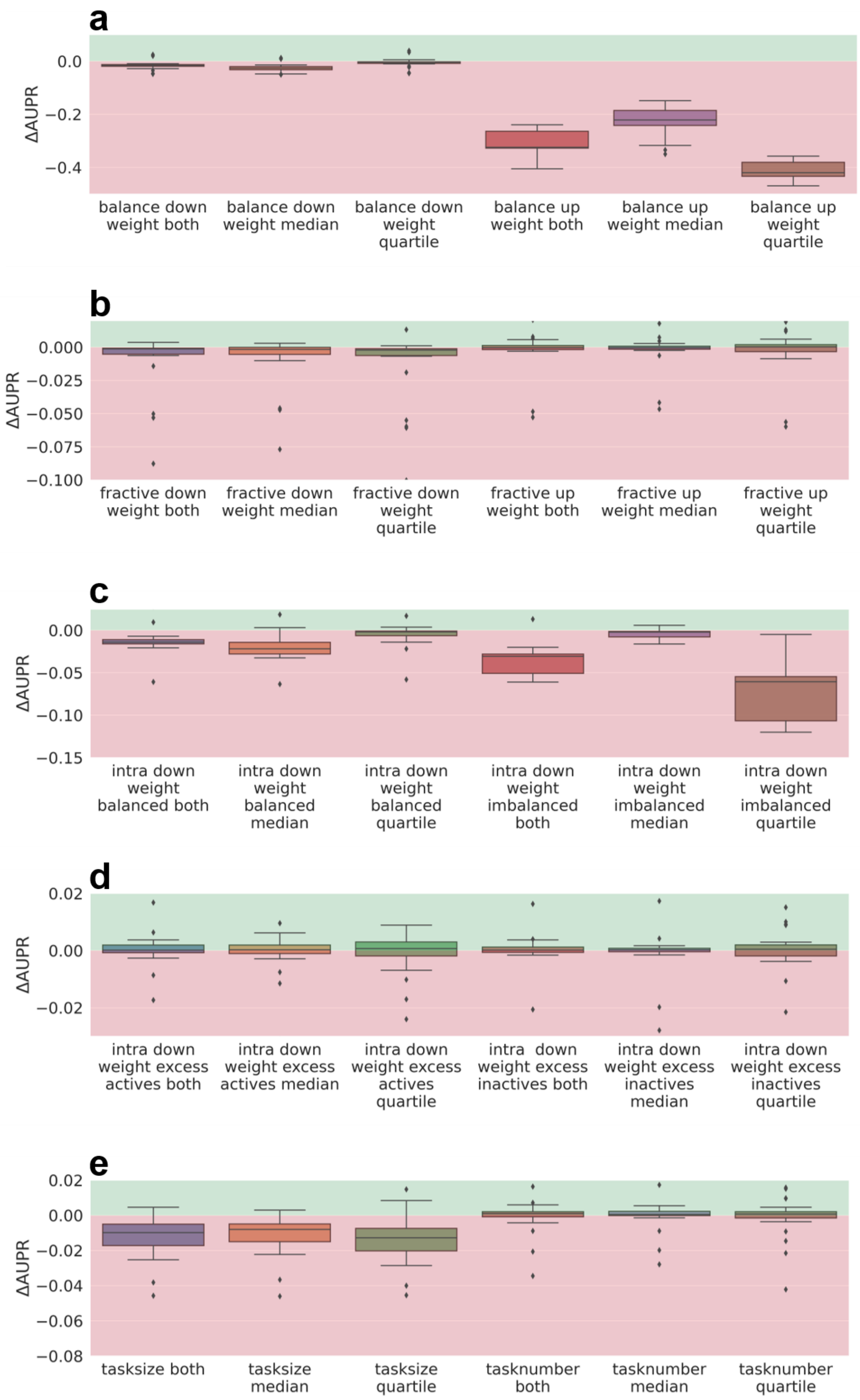

a
0.0
−0.2
−0.4
ΔAUPR
balance down weight both
balance down weight median
balance down weight quartile
balance up weight both
balance up weight median
balance up weight quartile
b
0.000
−0.025
−0.050
−0.075
−0.100
ΔAUPR
fractive down weight both
fractive down weight median
fractive down weight quartile
fractive up weight both
fractive up weight median
fractive up weight quartile
c
0.00
−0.05
−0.10
−0.15
ΔAUPR
intra down weight balanced both
intra down weight balanced median
intra down weight balanced quartile
intra down weight imbalanced both
intra down weight imbalanced median
intra down weight imbalanced quartile
d
0.02
0.00
−0.02
ΔAUPR
intra down weight excess actives both
intra down weight excess actives median
intra down weight excess actives quartile
intra down weight excess inactives both
intra down weight excess inactives median
intra down weight excess inactives quartile
e
0.02
0.00
−0.02
−0.04
−0.06
−0.08
ΔAUPR
tasksize both
tasksize median
tasksize quartile
tasknumber both
tasknumber median
tasknumber quartile

Figure S5. Phase III results averaged over 5 partners and 5 folds for synoptic and deconvoluted performances (median and lower quartile (blue and red boxes), only median (orange and purple boxes) and only lower quartile task (green and brown boxes), AUPR): (a) global weighting wrt. label balance left: down-weighting balanced tasks and right: down-weight imbalanced tasks, (b) global weighting wrt. fraction actives left: down-weighting excess of actives and right: down-weight excess of inactives, (c) intra assay weighting wrt. label balance left: down-weighting balanced tasks and right: down-weight imbalanced tasks, (d) intra assay weighting wrt. fraction actives left: down-weighting excess of actives and right: down-weight excess of inactives, (e) left: weight set wrt. number of datapoints and right: based on 1/task_number. Green: better performance than baseline (1), red: worse performance than baseline.
